# Diagnosis and treatment of primary soft tissue sarcomas: comprehensive review

**DOI:** 10.1093/bjsopen/zraf177

**Published:** 2026-03-09

**Authors:** Fahima Dossa, Eldad Elnekave, Aisha B Miah, Catherine Mitchell, Sarah Watson, Alessandro Gronchi, Marco Fiore

**Affiliations:** Department of Surgery, Cedars-Sinai Medical Center, Los Angeles, California, USA; Unit of Interventional Radiology, Shaare Tzedek Medical Center, Jerusalem, Israel; Sarcoma Unit, The Royal Marsden Hospital and the Institute of Cancer Research, London, UK; Department of Pathology, Peter MacCallum Cancer Centre and Sir Peter MacCallum Department of Oncology, University of Melbourne, Melbourne, Victoria, Australia; Department of Medical Oncology, Institut Curie Hospital, Paris, France; Sarcoma Service, Department of Surgery, Fondazione IRCCS Istituto Nazionale dei Tumori, Milan, Italy; Sarcoma Service, Department of Surgery, Fondazione IRCCS Istituto Nazionale dei Tumori, Milan, Italy

**Keywords:** sarcoma, retroperitoneal sarcoma, limb sarcoma, sarcoma guidelines, surgical treatment of sarcoma

## Abstract

**Background:**

Soft tissue sarcomas (STSs) comprise a group of rare malignancies with anatomic- and histologic-specific patterns of local and distant recurrence. Due to their rarity and histology-specific tumour behaviour, their natural history and the efficacy of various interventions may be challenging to assess. The aim of this review is thus to provide a comprehensive overview of diagnostic and treatment options for localized extremity and retroperitoneal STSs.

**Methods:**

A literature search was conducted to identify articles related to the diagnosis and management of localized extremity and retroperitoneal STSs. English-language articles published until June 2025 were identified using Medical Subject Heading terms on PubMed. Results were reported using a narrative approach. Topics highlighted in this review include diagnosis, institutional volumes, treatment options, and multimodal management of STSs based on location.

**Results:**

Accurate diagnosis of STS relies on carefully planned preoperative biopsies and in selected cases is supplemented by advanced molecular diagnostic tools. Surgery remains the cornerstone of curative-intent treatment for localized STSs; however, resectability criteria for retroperitoneal STSs vary by institution. Institutional case volumes are prognostic of outcome, with 10–20 retroperitoneal sarcoma cases per year considered by experts to be indicative of high-volume sarcoma centres. The role of adjunctive therapies, including chemotherapy, radiation, and/or other locoregional treatments, is dictated by histological and molecular characteristics associated with local and distant recurrence rates.

**Conclusion:**

The management of localized STSs is multidisciplinary in nature, requiring consideration of tumour and patient characteristics, and treatment factors. The rarity of STSs and the variable biological behaviour of the various histologic subtypes have impacted research in this field. Ongoing international collaborations and innovative study designs are essential for advancing the understanding of tumour behaviour and in optimizing treatment approaches.

## Teaser

This review outlines current diagnostic and treatment strategies for localized primary soft tissue sarcomas, with a separate focus on extremity/trunk and retroperitoneal disease. Key points include recent therapeutic advances, multidisciplinary management, and the centralization of care in high-volume centres to optimize outcomes.

## Introduction

Soft tissue sarcomas (STSs) comprise a histologically diverse group of rare tumours of mesenchymal origin. Accounting for < 1% of adult malignancies, most STSs arise sporadically with few identified genetic, immunologic, and environmental risk factors. Approximately 50% of STSs originate in the extremity or trunk, where well differentiated/dedifferentiated liposarcoma (WDLPS/DDLPS), myxoid/round cell liposarcoma, leiomyosarcoma, myxofibrosarcoma, and undifferentiated pleomorphic sarcoma (UPS) represent the major histologic subtypes, whereas about 15% develop in the retroperitoneum, predominated by WDLPS/DDLPS and leiomyosarcoma^1^. Tumour location, histologic subtype, and tumour grade are key prognostic factors, with higher-grade tumours demonstrating the poorest disease-free survival (DFS) and overall survival (OS)^1–3^. The rarity of STS has made focus on histology-specific evaluation challenging; however, previous studies^2,3^ demonstrate strong associations between histologic subtype and tumour behaviour, such as the propensity for local recurrence among retroperitoneal WDLPS, as opposed to the high risk of distant metastases present for similarly located leiomyosarcoma. Histology-specific tumour behaviour is particularly relevant when evaluating the effectiveness of available treatments. However, for practical purposes, previous studies often distinguish tumours by anatomic location rather than histology, complicating the understanding of the natural history of STS and the efficacy of various interventions. Herein, the aim was to provide a comprehensive summary of the diagnosis and current management of localized STS by anatomic site, highlighting nuances based on histologic variability, where data are available.

## Methods

A comprehensive review was conducted by a panel of international experts in STSs. To this end, English language articles published up to June 2025 were identified using Medical Subject Heading ‘sarcoma’ (classification, diagnosis, diagnostic imaging, epidemiology, mortality, pathology, radiotherapy, surgery, OR therapy) or the text word ‘soft tissue sarcoma’ on PubMed. The most relevant and recent literature for each area was reviewed, current scientific gaps were highlighted, and future directions were discussed. The review was divided into sections starting with a revision of current guidelines and addressing diagnosis, the impact of Institutional volumes, treatment options, and multimodal management of STSs according to their location (extremities and retroperitoneal disease), including local treatments. Guidelines and consensus statements on the workup and treatment of localized STSs were also retrieved and compared. To this end, the most recent version of the International guidelines in use in Western countries was retrieved and appraised. Results are reported using a narrative approach. Finally, future challenges arising in clinical practice and scientific research are discussed.

## Results

### STS guidelines

The review of STS guidelines included those published by the European Society for Medical Oncology (ESMO), the Transatlantic Australasian Retroperitoneal Sarcoma Working Group (TARPSWG), the National Comprehensive Cancer Network (NCCN), and those from the UK. The recommendations revealed a general consensus among recommendations for referral to high-volume/specialized centres, principles of biopsy and pathologic assessment, staging imaging, and recommendations for extent of surgery and use of neoadjuvant/adjuvant radiation and chemotherapy^4–7^. A summary of relevant guideline recommendations and consensus statements is provided in *Table 1*, and individual elements are discussed below.

**Table 1:** Comparison of guidelines and consensus statements regarding diagnosis and treatment of localized STS

ESMO (Gronchi *et al*. 2021)^5^	UK (Hayes *et al*. 2025)^6^	NCCN (2025)^4^	TARPSWG (Swallow *et al*. 2021)^7^
**Treatment at specialized centres**			
Management should be carried out in sarcoma reference centres or tertiary paediatric oncology centres, as appropriate for age. Patients with suspected RPS need to be referred to high-volume sarcoma centres.	All patients with a suspected STS should be managed by a specialist sarcoma MDT as specified in the NICE guidance. Patients with retroperitoneal/intra-abdominal masses suspicious for sarcoma should be referred to a specialist MDT before biopsy or surgical treatment.		Volume-outcome relationships in the surgical care of RPS support the regionalization of care to high-volume hospitals (10–20 RPS cases/year). The MDT that makes decisions should include a surgeon with specialized training in resection of RPS. The decision-making team should also include a radiologist, pathologist, medical oncologist, and radiation oncologist with a practice focused on caring for patients with RPS.
**Biopsy**			
	A pretreatment histopathological diagnosis should be made, if possible, by percutaneous core biopsy. The optimal management of RPS is facilitated by pretreatment diagnosis and an image-guided percutaneous CNB is strongly recommended. Laparotomy and open biopsy or laparoscopic biopsies of suspected RPS should be avoided.	A pretreatment biopsy (CNB) is highly preferred for the diagnosis and grading of STS. Biopsy should be performed by an experienced surgeon or radiologist, placed along the future resection axis with minimal dissection and careful attention to haemostasis.	An image-guided percutaneous coaxial CNB (14–18 gauge) is strongly recommended as the standard of care. Biopsy may occasionally be omitted if the imaging is judged pathognomonic (for example, heterogeneous WDLPS/DDLPS) by an expert radiologist within an expert multidisciplinary tumour board, and no preoperative treatment is planned. Sampling of the more solid tumour component represented by well perfused areas on contrast-enhanced CT or MRI is encouraged to avoid undergrading, as these areas are more likely to represent high-grade/dedifferentiated disease. Elective surgery for resection of an RP tumour without preoperative biopsy, without referral to a specialist centre, and/or without multidisciplinary tumour board discussion is strongly discouraged. Laparotomy and open biopsy (and laparoscopic biopsy) of suspected RPS should be avoided.
**Pathology**			
Pathological diagnosis should be made by a sarcoma expert pathologist according to the 2020 WHO classification.	Biopsy should be reviewed by a specialist sarcoma pathologist for diagnostic confirmation and appropriate molecular and genomic analysis.	Pathologists with expertise in STS should review the pathologic assessment of biopsies and resected specimens, especially for initial histopathological classification. Margins must be thoroughly evaluated in these specimens. Morphologic assessment based on microscopic examination of histologic sections remains the standard of sarcoma diagnosis. Molecular testing should be performed by a pathologist with expertise in the use of molecular diagnostic techniques for the diagnosis of STS. In addition, technical limitations associated with molecular testing suggest that molecular evaluation should be considered only as an ancillary technique.	A selective approach should be applied to testing for specific translocations to elucidate the histologic subtype.
**Extremity STS**			
Imaging and staging			
Staging is routinely carried out with contrast-enhanced chest, abdomen, and pelvis CT. Whole-body MRI may be an alternative, especially in selected histotypes. Brain CT/MRI may be indicated only in ASPS, CCS, and angiosarcoma. FDG-PET is indicated as a problem-solving tool in equivocal cases.	Cross-sectional imaging of the primary tumour, usually in the form of MRI, is recommended before definitive surgery. Imaging of the thorax by CT scan for lung metastases should be performed before radical treatment. Further staging may be considered depending on subtype and location of the sarcoma.	Recommendation for MRI with contrast, with or without CT with contrast. Given the risk of haematogenous spread from a high-grade sarcoma to the lungs, imaging of the chest (CT without contrast (preferred) or X-ray) is essential for accurate staging. Abdominal/pelvic CT should be considered for angiosarcoma, leiomyosarcoma, myxoid/round cell liposarcoma, or epithelioid sarcoma as well as STS without definitive pathology before final resection. MRI of the total spine should be considered for myxoid/round cell liposarcoma due to the higher risk of metastasis to the spine compared with other STSs. CNS MRI should be considered for patients with alveolar soft part sarcoma and angiosarcoma.	
Surgery			
Surgery is the standard treatment for all patients. It must be carried out by a surgeon specifically trained in the treatment of STSs. The standard surgical procedure is an *en bloc* wide excision with R0 margins.	Surgery is the standard treatment for most patients with localized STS. For patients with resectable disease, a wide excision through normal uninvolved tissues is the surgical procedure of choice. With the addition of adjuvant RT, a close but tumour-free margin (R0) may be adequate. Where a wide excision is not possible due to anatomical constraints, a planned marginal or microscopically positive margin against a critical structure, plus RT, for intermediate- and high-grade tumours, may be an appropriate means of achieving tumour control while maintaining physical function.	Limb-sparing surgery is recommended for most patients with STS of extremities to achieve local control with minimal morbidity. Surgical resection (with appropriately negative margins) is the standard primary treatment for most patients with STS, although close margins may be necessary to preserve uninvolved critical neurovascular structures. Radical excision or entire anatomic compartment resection is not routinely necessary.	
Radiation			
Wide excision and RT are the standard treatments for high-grade (G2–3) lesions. The sequence of the two treatments varies among institutions, but there is an overall shift towards the use of preoperative RT, especially when preserving a critical structure is one of the goals. RT can be omitted only after multidisciplinary discussion in reference centres considering several variables.	For patients with borderline resectable tumours, preoperative treatment with chemotherapy and/or RT should be considered depending on histology. Pre- or postoperative RT is recommended along with surgical resection of the primary tumour for the majority of patients with intermediate- and high-grade tumours, and for selected patients with large or marginally excised, low-grade tumours.	RT and/or chemotherapy are often used before surgery to downstage large high-grade tumours to enable effective surgical resection. Postoperative RT should be considered following resection with close soft tissue margins (<1 cm) or a microscopically positive margin on bone, major blood vessels, or a nerve. Because postoperative radiation fields are typically larger than preoperative fields, the panel has expressed a general preference for preoperative radiation, particularly when treatment volumes are large.	
Chemotherapy and other adjunctive treatments			
Adjuvant/neoadjuvant anthracycline–ifosfamide chemotherapy for at least three cycles can be proposed to patients at high risk of death. Neoadjuvant chemotherapy with regional hyperthermia is another individualized option in patients at high risk of death	For patients with borderline resectable tumours, preoperative treatment with chemotherapy and/or RT should be considered depending on histology.Neoadjuvant or adjuvant chemotherapy is not routinely recommended but should be considered in situations where achieving local control is likely to be compromised, or the prognosis is poor, particularly in more chemosensitive sarcoma subtypes. Risk-stratification can be performed using nomograms such as Sarculator where patients with 5-year predicted survival < 60% may be most likely to benefit.	RT and/or chemotherapy are often used before surgery to downstage large high-grade tumours to enable effective surgical resection.	
**Retroperitoneal STS**			
Imaging and staging			
		CT is the preferred imaging modality, although MRI can also be utilized in certain situations. Chest imaging should be performed for histologies that have the potential for lung metastases.	Thorough review of cross-sectional imaging by a sarcoma tumour board is required. The standard method for staging for the extent of primary tumour and for distant metastases is CT of the chest/abdomen/pelvis with i.v. contrast. A baseline PET scan may be considered before the treatment of high-grade RPS, but is not regarded as essential.
Intraoperative suspicion for RPS			
		If a retroperitoneal STS is encountered unexpectedly when a laparotomy is performed for some other reason, a CNB should be performed to establish the diagnosis as well as the histopathologic type and grade of the tumour.	If at open or laparoscopic exploration for suspected adnexal mass no abnormalities of the uterus, fallopian tubes, or ovaries are found but an RP mass is detected, it is recommended that nothing further be done at that time. The patient should undergo subsequent dedicated imaging and referral to a sarcoma referral centre.
Surgery			
Standard treatment of RPS consists of surgical resection *en bloc* with adherent organs.	The standard of care is *en bloc* macroscopically complete resection of the tumour and involved/adjacent organs, performed in high-volume specialist sarcoma centres. In the case of retroperitoneal liposarcoma, surgery to resect the tumour and adjacent viscera, irrespective of involvement and clearing all ipsilateral fat, to minimize microscopic positive margins, should be considered. Resection often necessitates ipsilateral nephrectomy, hemicolectomy, psoas fascia/muscle resection, and distal pancreatectomy/splenectomy on the left.		Complete *en bloc* gross resection is the cornerstone of management. In the case of primary RPS, surgery should be aimed at achieving macroscopically complete resection, with a single specimen encompassing the tumour and involved contiguous organs. Given the uncertainty regarding margin definition, an extended approach to systematically resect adherent viscera, irrespective of expected microscopic infiltration, should be considered for retroperitoneal liposarcoma.
Radiation			
Neoadjuvant RT has shown signs of efficacy in primary low/intermediate-grade retroperitoneal liposarcoma. Intraoperative/postoperative RT is of no proven value in RPS.	Abdominal recurrence-free survival is significantly improved in the low–intermediate grade liposarcoma subgroup (with administration of preoperative RT) and preoperative RT should be discussed with this group. Postoperative RT following complete resection is of limited value and associated with significant toxicities and should only be considered in selected cases with a well defined area at risk of local recurrence.	Neoadjuvant RT can be considered for selected patients with retroperitoneal/intra-abdominal STS who are at high risk for local recurrence. The panel discourages adjuvant RT for retroperitoneal/intra-abdominal STS except in highly selected cases where local recurrence would cause undue morbidity.	Routine use of neoadjuvant RT is not recommended in patients with high-grade RPS, but may be considered in those with high risk of local (abdominal)-only recurrence, that is WDLPS and low-grade DDLPS. Postoperative/adjuvant external beam radiation after complete gross resection is of no proven benefit and is associated with significant short- and long-term toxicities.
Chemotherapy			
The role of adjuvant/neoadjuvant chemotherapy is not yet established.	Preoperative chemotherapy can be considered for chemo-sensitive subtypes such as synovial sarcomas and borderline resectable leiomyosarcoma. The value of adjuvant chemotherapy is not established and cases at high risk for metastatic disease should be individually discussed. The Sarculator nomogram can be used for prognostication.		Postoperative/adjuvant chemotherapy after complete gross resection is of no proven benefit. Neoadjuvant chemotherapy can be discussed for use in individual patients with chemosensitive histologies such as synovial sarcoma and high-grade LMS, among others, or within prospective clinical studies.

STS, soft tissue sarcoma; ESMO, European Society for Medical Oncology; NCCN, National Comprehensive Cancer Network; TARPSWG, Transatlantic Australasian Retroperitoneal Sarcoma Working Group; RPS, retroperitoneal sarcoma; MDT, multidisciplinary tumor board; NICE, National Institute for Health Care Excellence; CNB, core needle biopsy; WDLPS, well differentiated liposarcoma; DDLPS, dedifferentiated liposarcoma; CT, computerized tomography; RP, retroperitoneal; WHO, World Health Organization; ASPS, alveolar soft part sarcoma; CCS, clear cell sarcoma; FDG-PET, fluorodeoxyglucose-positron emission tomography; CNS, central nervous system; RT, radiation therapy/radiotherapy; i.v., intravenous; LMS, leiomyosarcoma.

### Diagnostic considerations for STS

#### Local staging

The diagnostic pathway for STS begins with imaging of the primary site to assess the mass’s size, location, composition, and relationship to neighbouring organs and neurovascular structures^8^. For extremity and trunkal STS, magnetic resonance imaging (MRI) is the preferred modality for assessing local extent, including signal intensity on T1- and T2-weighted images, contrast enhancement patterns, and fat-saturated sequences, and provides insight into the suspected histologic subtype and informs treatment planning. MRI can also serve as the primary local imaging modality for pelvic tumours. For most retroperitoneal and intra-abdominal tumours, computerized tomography (CT) with intravenous contrast is preferred for assessment of local disease and involvement of visceral and vascular structures, with arterial phases used, as needed, for surgical planning^7^. Nodal basin-directed imaging is typically not required due to the low likelihood of nodal metastases secondary to the primarily haematogenous spread of sarcomas, except in specific subtypes with known proclivity for nodal metastases (that is, angiosarcoma, clear cell sarcoma, epithelioid sarcoma, and rhabdomyosarcoma).

#### Biopsy and histopathological assessment

Whereas select tumours demonstrate pathognomonic imaging features allowing for reliable diagnosis (for example, angiomyolipoma), radiologic assessment alone is typically insufficient for diagnosis of STS. In a study^9^ of 291 patients with retroperitoneal sarcoma (RPS), standardized radiologic assessment demonstrated only moderately favourable performance characteristics for diagnosing mesenchymal adipocytic tumours (sensitivity 79.1%, specificity 99.4%) and poor discrimination for non-adipocytic tumours (sensitivity 55.4%, specificity 0%). Biopsy is, therefore, strongly recommended. An exception can be made for retroperitoneal tumours judged by an expert sarcoma radiologist as demonstrating features highly suggestive of WDLPS if no preoperative treatment is planned and this decision is agreed upon by an expert multidisciplinary tumour board^7^. For extremity masses, biopsy can be omitted for superficial, purely adipocytic tumours measuring ≤ 10 cm, with no radiologic features suspicious for malignancy, often classified as atypical lipomatous tumours^8^. Biopsy is particularly important for patients being considered for preoperative therapy, where histologic subtype or tumour grade may dictate treatment eligibility or sequencing, as it can impact treatment choice and/or timing in up to 46% of cases^10^.

A percutaneous core needle biopsy (CNB) is the preferred approach for tissue sampling, with high accuracy for ascertaining histologic subtype (> 80% concordance with final pathology^11–14^). However, accuracy for grade via biopsy is lower (60 and 70%^15–18^), often due to undergrading of the biopsy specimen. This is exemplified by the common misclassification of DDLPS as WDLPS, often due to sampling error^11,15,16^ but can be observed in cases of leiomyosarcoma as well^17^. Intratumoural heterogeneity, especially for tumours comprised of both well differentiated and dedifferentiated liposarcomatous components, contributes to this issue. As such, Tirotta *et al.*^19^ have demonstrated that targeting solid areas of tumours on biopsy can improve sensitivity for identifying DDLPS. This practice is further supported by the TARPSWG guidelines, which recommend tissue sampling from solid, well perfused components on contrast-enhanced CT or MRI or from high standardized uptake value areas on fluorodeoxyglucose-positron emission tomography/CT, if performed, to increase the probability of detecting high-grade disease^7^. Whereas sampling from solid, non-necrotic regions can improve the accuracy of grading, degree of necrosis is a component of tumour grading, and, as such, correlating pathologic assessment with radiographic features in highly necrotic tumours may aid in accurate preoperative estimation of tumour grade.

A CNB carries low risks. Early complications occur in about 3% of cases^10,11,15^, most commonly bleeding or pain. Needle tract seeding has been a theoretical concern, as malignant cells were found to be present in 13% of needle tracts excised in the context of musculoskeletal sarcomas^20–23^. However, pooled results of two studies^20,24,25^ in which biopsy tracts were not excised showed no events consistent with needle tract seeding. This suggests that the presence of malignant cells alone is likely insufficient to allow for tumour growth. In the retroperitoneum, seeding rates are estimated at ≤ 0.5%^10,11,26,27^, rising to 2.0% in one study involving non-coaxial transabdominal biopsies^28^. Concerns regarding biopsy-related tumour pseudocapsule breach compromising oncologic outcomes have also been investigated, with studies^26,28^ showing no increase in local recurrence when a CNB is performed.

**Fig. 1:**
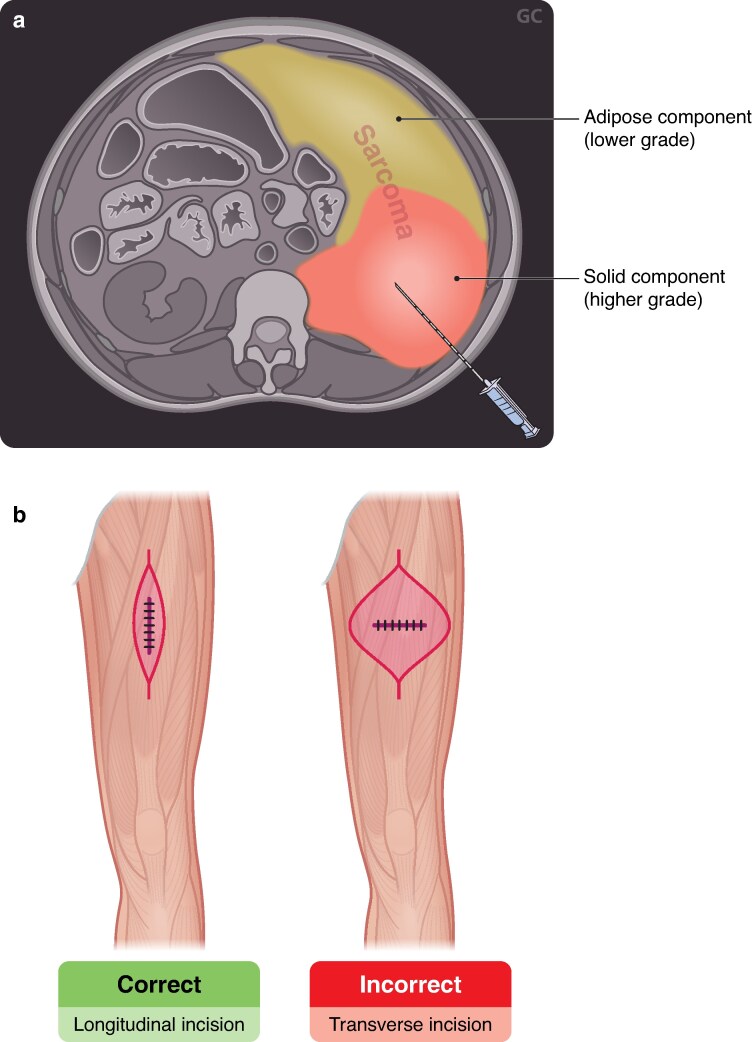
Biopsy techniques for a retroperitoneal soft tissue sarcoma approached with retroperitoneal biopsy with coaxial needle technique **a** Image-guided biopsy can target the highest-grade areas of the tumour. The retroperitoneal, rather than transabdominal, approach reduces the potential risk of intra-abdominal seeding. Additionally, the confined retroperitoneal space provides tamponade in cases of post-biopsy bleeding. **b** Extremity soft tissue sarcoma approached with incisional/excisional biopsy. Longitudinal, as opposed to transverse, biopsy prevents contamination of uninvolved fascial compartments and reduces the need for unnecessary complex skin and soft-tissue coverage at the time of definitive resection.

To minimize complications and avoid compromising future surgery, best practices for a CNB are recommended. These include use of a 14–18 gauge needle via a coaxial technique with four to six passes, targeting higher-density/solid areas, to maximize diagnostic yield^7,19,29,30^. Within the extremity, the biopsy site should be placed such that the tract can be easily excised *en bloc* with the specimen at the time of definitive surgery. For retroperitoneal tumours, where the biopsy tract is not typically excised, retroperitoneal approaches to biopsy should be favoured over transabdominal routes to minimize the risk of seeding (*Fig. 1*, panel A).

A CNB may not always be feasible and may require consideration of alternative biopsy techniques. Small (< 3 cm) superficial extremity/truncal lesions may be best sampled via excisional biopsy^5,8^; however, this method is generally avoided for larger lesions as re-excision for residual disease may necessitate creation of a large defect with associated functional losses^12^ and preclude patients from neoadjuvant therapies. Incisional biopsy, once common, is now infrequently performed, as complication rates, including haematoma, infection, and wound dehiscence, are higher than for a CNB. However, it may be considered in select cases discussed at referral centres^5^. When performed, incisions should be made longitudinally in the extremities to minimize the extent of soft tissue resection required at definitive surgery (*Fig. 1*, Panel B). Dissection should avoid uninvolved tissues and attempt not to violate fascial planes. Meticulous haemostasis is additionally important, as post-biopsy haematomas, considered contaminated by malignant cells, can dissect through tissues, further contaminating initially uninvolved compartments^29,31^. Laparotomy or laparoscopy for biopsy should generally not be performed, as a lack of image guidance can lead to undersampling, risks of peritoneal contamination, and distorted planes for future surgery^7^. Similarly, when a retroperitoneal mass is unexpectedly encountered during surgery, then surgery should be aborted, and postoperative imaging and percutaneous biopsy pursued^7^.

Histopathological diagnosis is typically made using morphology and immunohistochemistry, with molecular diagnostics offering valuable adjuncts. These tests leverage known genetic alterations in various sarcomas, including translocations, specific oncogenic mutations, and gene amplifications or deletions,^32^ such as *MDM2* amplification in WDLPS/DDLPS, as detected by fluorescence *in situ* hybridization^33^. GENSARC^34^, a prospective study of the utility of molecular assays for sarcoma diagnosis, demonstrated that routine molecular genetic testing can modify diagnoses in up to 23% of cases. As such, molecular diagnostic adjuncts are recommended for specific histologic subtypes that require molecular confirmation for diagnosis; when there is diagnostic uncertainty, including cases of unusual clinical presentations or discordance between clinical and pathological appearances; for subtypes where specific diagnoses will influence use of neoadjuvant therapeutic strategies (for example, synovial sarcoma, myxoid/round cell liposarcoma, and Ewing’s family sarcoma); or for prognostication or prediction^5^.

Radiomics is an emerging adjunctive tool under investigation to improve the ascertainment of histologic subtype and grade. Although promising results are emerging, these methods remain investigational at this time.

#### Distant staging

Following diagnostic confirmation and evaluation of local extent of disease, distant staging is required. Sarcomas typically spread via haematogenous routes. Given the predilection for pulmonary metastases in extremity sarcomas, distant staging is usually performed with chest CT. Abdominopelvic imaging is additionally performed for histologic subtypes with a proclivity for intra-abdominal or retroperitoneal metastases, including angiosarcoma, leiomyosarcoma, myxoid/round cell liposarcoma, and epithelioid sarcoma^4^. Further histology-specific patterns of spread should also be considered. Some examples include the various sites of extrapulmonary metastases possible in myxoid liposarcoma (intra-abdominal/retroperitoneal, osseous, truncal, cardiac/mediastinal)^35,36^ and the risk of brain metastases in alveolar soft part sarcoma^5^. Distant staging for RPS includes thoracic, abdominal, and pelvic CT.

### Importance of high-volume centres

Current guidelines^5,7^ recommend that patients with STS be managed within designated sarcoma referral centres or networks. The rationale for these recommendations comes from numerous studies demonstrating improved outcomes when patients with sarcoma are treated at high-volume centres, including greater guideline-concordant care^37–39^, greater R0 and fewer R2 resections^37,38,40–43^, and improved survival outcomes^37,39,42,44–48^. Thresholds for defining high-volume hospitals vary across studies; however, a US study^49^ using data from the National Cancer Database suggested improved survival for RPS with increasing hospital case volume, which plateaued at 13 cases/year. This threshold has subsequently been used to update sarcoma nomograms to incorporate volume–outcome relationships^45^. Surveyed members of TARPSWG similarly suggested RPS case volumes of 11–20 per year as indicative of high-volume RPS centres^49^. Importantly, although guidelines recommend treatment at referral centres, many patients with sarcoma continue to be treated in low-volume hospitals that manage > 90% of patients with sarcoma^42^. Although this proportion varies by jurisdiction, with some countries reporting that over half of patients are treated in high-volume hospitals, the rate of regionalization adoption is slower than that observed in other cancers, such as pancreatic cancer^50^.

Critically, hospital case volume alone does not entirely account for improved outcomes observed in high-volume hospitals. In fact, in one study, as the number of cases of RPS treated at community hospitals increased, mortality also increased—a trend not seen in academic hospitals^51^. Such data illustrate that the superior outcomes attained at high-volume centres are attributable to systems of care, comprising multidisciplinary teams (including experts in sarcoma radiology and pathology), resources to deliver guideline-concordant treatments, and patient access to clinical and translational research studies—all factors recommended as quality criteria for sarcoma reference centres^5,52^. For example, patients who present to multidisciplinary tumour boards at sarcoma reference centres before initiation of therapy are more often guideline-concordant and have higher rates of R0 resections^53^. The development of the TARPSWG international tumour board^54^ is one example of an innovation seeking to extend these benefits to the broader sarcoma community; however, as detailed above, care administered at high-volume centres capitalizes on several processes associated with improved outcomes and should be pursued, when possible.

Within high-volume centres, individual surgeon case volumes may also influence outcomes. Only one study^55^ has evaluated the learning curve for RPS surgery based on operative metrics for a single surgeon, demonstrating a plateau in improvements in operative time after 16 cases and a post-learning phase signalled by greater case complexity after 46 cases; however, studies^56^ in other cancer types emphasize an interplay between hospital and surgeon case volumes on surgical morbidity and mortality. Together, these highlight opportunities for both regional and local centralization of sarcoma surgical expertise to optimize patient outcomes.

### Treatment of extremity STS

#### Surgery

The goal of surgery for extremity STS is a limb-sparing, function-preserving, margin-negative resection. The foundation for this approach was laid by the seminal trial by Rosenberg *et al*.^57^, which randomized patients to amputation, the previously favoured approach, or limb-sparing resection with adjuvant radiation therapy/radiotherapy (RT); patients in both groups additionally received adjuvant chemotherapy. Although patients randomized to the limb-sparing arm had higher rates of local recurrence, DFS and OS were comparable to those with amputation, leading to the acceptance of limb-sparing resection as the standard of care.

Appropriate limb- and function-preserving surgery requires a detailed understanding of histotype-specific recurrence patterns and functional anatomy, the latter aided by a thorough review of high-resolution imaging for surgical planning^6^. Importantly, malignant cells frequently extend beyond the tumour pseudocapsule^58^ and, as such, wide resections (into surrounding uninvolved tissue) are recommended over marginal excisions (extending just outside the pseudocapsule). An exception to this rule is made for atypical lipomatous tumours, for which marginal excision may be appropriate, given the low recurrence rate even with positive margins^5,59^. Surgical planning should consider whether skin or soft-tissue coverage will be required, necessitating collaboration with an oncoplastic surgeon.

Where extremity STSs abut critical neurovascular structures, planned R1 resections are justified to preserve these structures^5^. Several studies demonstrate low rates of local recurrence in the setting of planned R1 resections, compared with the high rates observed with unexpected positive margins^59,60^. However, if critical vasculature or nerves are encased, resection and reconstruction is feasible, particularly when required to facilitate limb salvage. Arterial reconstruction can be accomplished with synthetic or autologous grafts. Whereas acceptable functional outcomes may be achievable with resection of select major nerves^61–63^, reconstructive techniques, when possible, can restore some sensory and motor function^64^. Contemporary cohorts estimate amputation rates of < 5%. Indications for primary amputations have included multifocal disease, bone invasion, neurovascular bundle involvement with loss of function, and comorbidities precluding major reconstructive surgery^65,66^.

A not infrequent scenario encountered by the sarcoma surgeon is referral of a patient after marginal excision of a presumed benign mass elsewhere, with final pathology unexpectedly demonstrating sarcoma. When such patients undergo re-excision to obtain wider margins, residual malignancy is identified in 24–63% of specimens^67,68^. On initial consultation, such patients should be appropriately staged, including imaging of the involved limb to rule out gross residual disease^6^. In the absence of radiologically evident disease, re-excision at a sarcoma centre can be considered to optimize local control if it can be achieved without excessive morbidity^5,6^. However, systematic re-excision is not associated with improvements in metastasis-free or OS and, as such, watchful waiting with delayed re-excision at the time of local recurrence is an alternate option^69^.

#### Radiation

RT is used adjunctively in the treatment of extremity STS to improve local control. A synthesis of the evidence is provided in *Table 2*. The basis for this approach arises from Rosenberg *et al*.’s seminal paper, which introduced external beam radiotherapy (EBRT) following function-preserving surgery as an alternative to amputation in 43 patients in a randomized clinical trial (RCT)^57^. The limb-sparing resection group received wide local excision followed by 50 Gy in 25 fractions to the entire anatomic area at risk for local spread (commonly 5 cm longitudinal margin and 2 cm axial margin) and 60–70 Gy (additional 10–20 Gy at 2 Gy per fraction) to the tumour bed. There were four recurrences in the limb-sparing group associated with marginal resection compared with zero recurrences in the amputation group. Further, in the National Cancer Institute study by Yang *et al.*^70,71^ patients with extremity STS were randomized to receive or forgo adjuvant EBRT; all patients with high-grade disease additionally received adjuvant chemotherapy. This trial demonstrated improvements in local control with adjuvant RT, with benefits observed in both low- and high-grade disease. Neither study found improvements in rates of distant recurrence or survival, thus concluding that function-preserving surgery with EBRT was an acceptable standard of care.

**Table 2:** Seminal trials in RT and systemic therapy for eSTS

Study	Population	Interventions	Outcomes	Conclusions
**RT**				
Adjuvant external beam RT				
Rosenberg *et al*. (1982)^57^	High-grade eSTS (*n* = 43).	Amputation *versus* limb-sparing resection + adjuvant RT (both groups received adjuvant chemotherapy).	Increased local recurrence with limb salvage (15% *versus* 0%; *P* = 0.06). No difference in 5-year DFS between limb salvage *versus* amputation (71% *versus* 78%; *P* = 0.75). No difference in 5-year OS between limb salvage *versus* amputation (83% *versus* 88%; *P* = 0.99).	Limb-sparing surgery + adjuvant RT is a safe alternative to amputation.
NCI trial (Yang *et al*. 1998)^70^	Low-grade (*n* = 50) and high-grade (*n* = 91) eSTS.	Low-grade: surgery +/− adjuvant RT. High-grade: surgery + adjuvant chemotherapy +/− adjuvant RT.	High-grade: decreased local recurrence with adjuvant RT (*P* = 0.003) with no difference in OS. Low grade: decreased local recurrence with adjuvant RT (*P* = 0.02) with no difference in OS.	Adjuvant RT improves local control but not survival.
Neoadjuvant *versus* adjuvant RT				
NCIC SR2 trial (O’Sullivan *et al*. 2002, Davis *et al*. 2005)^72,73^	eSTS (*n* = 190).	Preoperative RT (50 Gy in 25 fractions) *versus* postoperative RT (66 Gy in 33 fractions).	Higher rate of wound complications in the preoperative RT *versus* postoperative group (35.2% *versus* 17.0%). Higher rate of long-term joint fibrosis in the postoperative *versus* preoperative RT group (48.2% *versus* 31.5%).	Preoperative RT associated with greater risk of early wound complications (particularly for lower extremity lesions), whereas postoperative RT associated with higher risk for long-term joint complications.
Adjuvant brachytherapy				
Pisters *et al*. (1996)^74^	Extremity or superficial trunk STS (*n* = 164).	Surgery alone *versus* surgery plus adjuvant brachytherapy (iridium-192 implant; 42–45 Gy over 4–6 days)	Improved 5-year local control with brachytherapy (82% *versus* 69%; *P* = 0.04). Effect limited to patients with high-grade disease (89% *versus* 66%; *P* = 0.003). No significant difference in distant recurrence or disease-specific survival.	Adjuvant brachytherapy can improve local control in high-grade disease, with no improvement in survival.
IGRT				
RTOG-0630 phase II trial (Wang *et al*. 2015)^75^	eSTS (*n* = 79).	Preoperative IGRT followed by limb-sparing resection.	Grade ≥ 2 toxicity = 10.5% (historic comparator: 36.4% from NCIC-SR2 study). Wound complication rate = 36.6%.	Reduction of late but not early toxicity with IGRT.
Hypofractionated RT				
HYPORT-STS phase II trial (Guadagnolo BA *et al*. 2022, Bishop AJ *et al*. 2025)^76,77^	Extremity or superficial trunk STS (*n* = 120).	Preoperative moderately hypofractionated RT (15 fractions × 2.85 Gy).	Four-year local recurrence-free survival = 93%. Major wound complications = 30.8%. Two-year grade ≥ 2 toxicity = 9.1%. Bone fractures = 3.3%.	Suggests safety and efficacy of moderately hypofractionated RT (biologically equivalent dose 49 Gy).
Kosela-Paterczyk H *et al*. (2014)^78^	Extremity or truncal STS (*n* = 272).	Preoperative ultra-hypofractionated RT (5 fractions × 5 Gy).	Local recurrence = 19.1% (median f/u 35 months). Treatment toxicity (any grade) = 41.9%.	Compromised local control when biologically equivalent dose is lower than conventional RT (37.5 Gy).
Kalbasi A *et al*. (2020)^79^	Extremity or truncal STS (*n* = 52) planned for neoadjuvant RT.	Preoperative ultra-hypofractionated RT (5 fractions × 6 Gy).	Major wound complications = 32%. Grade ≥ 2 toxicity = 16%.	Ultra-hypofractionated regimens can demonstrate similar acute wound complication rates as conventionally fractionated RT when a biologically equivalent dose similar to conventional RT is used (50 Gy).
Leite ETT *et al*. (2021)^80^	eSTS (*n* = 25).	Preoperative SABR (40 Gy in 5 fractions administered every other day).	Wound complications = 28%. Grade ≥ 2 late toxicity = 41%. 2-year local recurrence = 0%. Amputation rate = 16%, fracture = 4%.	High amputation rate at high biologically equivalent doses (80 Gy).
Bedi M *et al*. (2022)^81^	Extremity or truncal STS (*n* = 32).	Preoperative ultra-hypofractionated RT (5 fractions × 7 Gy).	Local recurrence = 0% (median follow-up 36.4 months). Major wound complications = 25%. Amputation rate = 6%.	Acceptable local control at biologically equivalent dose similar to conventional RT (64 Gy).
Mayo ZS *et al*. (2023)^82^	Extremity and truncal STS (*n* = 22).	Preoperative ultra-hypofractionated RT (5 fractions × 6 Gy).	Local recurrence = 0% (median follow-up 24.5 months). Major wound complication = 41%. Wound complications requiring reoperation = 36%. Fracture rate = 5%.	High wound complication and fracture rates even at biologically equivalent dose similar to conventional RT (50 Gy).
**Systemic therapy**				
Chemotherapy				
Meta-analysis of RCTs (Pervaiz *et al*. 2008)^83^	RCTs of adjuvant chemotherapy for localized STS (*n* = 18 studies).	Adjuvant chemotherapy *versus* surgery alone.	Decreased odds of local recurrence with chemotherapy (OR 0.73, 95% c.i. 0.56, 0.94). Improved odds of survival with combination doxorubicin + ifosfamide *versus* no chemotherapy (OR 0.56, 95% c.i. 0.36, 0.85).	Marginal efficacy of chemotherapy for resectable STS that needs to be balanced against toxicity.
EORTC 62 931 (Woll *et al*. 2012)^84^ and *post hoc* analysis (Pasquali *et al*. 2019)^85^	STS of any site (*n* = 351).	Adjuvant chemotherapy (doxorubicin + ifosfamide) or no chemotherapy.	No difference in OS (HR 0.94, 95% c.i. 0.68, 1.31). No difference in relapse-free survival (HR 0.91, 95% c.i. 0.67, 1.22). *Post hoc* analysis: improvement in DFS (HR 0.49, 95% c.i. 0.28, 0.85) and OS (HR 0.50, 95% c.i. 0.30, 0.90) among high-risk patients (10-year predicted OS < 60%).	No benefit to chemotherapy in the overall population. High-risk patients (as determined by Sarculator-predicted 10-year OS < 60%) may benefit from chemotherapy.
ISG-STS 1001 (Gronchi *et al*. 2017, Gronchi *et al*. 2020)^86,87^	High-risk (high grade, ≥ 5 cm, deep-seated) extremity or truncal STS; inclusion of five histological subtypes (myxoid liposarcoma, leiomyosarcoma, synovial sarcoma, malignant peripheral nerve sheath tumour, undifferentiated pleomorphic sarcoma) (*n* = 287).	Three doses of neoadjuvant standard chemotherapy (epirubicin + ifosfamide) or histotype-tailored chemotherapy.	Stopped early for futility—improved 46-month DFS projected standard *versus* histotype-tailored chemotherapy arm (62% *versus* 38%; *P* = 0.004). In long-term follow-up, improved 5-year survival in standard *versus* histotype-tailored arm (76% *versus* 66%; *P* = 0.02).	When neoadjuvant chemotherapy is administered, standard regimens (not histotype-tailored regimens) are preferred.
Immunotherapy				
SARC028 (Tawbi *et al*. 2017)^88^	Advanced/metastatic soft tissue (*n* = 40) or bone sarcomas (*n* = 40) had received up to three lines of previous therapy.	Pembrolizumab 200 mg i.v. every 3 weeks.	Notable objective response rate: Undifferentiated pleomorphic sarcoma = 40% DDLPS = 20%.	Suggestion of efficacy of immunotherapy in undifferentiated pleomorphic sarcoma and DDLPS.
Alliance A091401 (D’Angelo *et al*. 2018)^89^	Advanced/metastatic sarcoma, at least one line of previous systemic therapy (*n* = 85).	Nivolumab 3 mg/kg every 3 weeks OR nivolumab 3 mg/kg plus ipilimumab 1 mg/kg every 3 weeks × 4 doses (independent, non-comparative arms).	Response rate: Nivolumab alone = 5%. Ipilimumab/nivolumab = 16%. Serious treatment-related adverse events: Nivolumab alone = 19%. Ipilimumab/nivolumab = 26%.	Nivolumab alone ineffective in advanced sarcoma. Combination ipilimumab/nivolumab may have efficacy but with increased toxicity.
Roland *et al*. (2024)^90^	Resectable DDLPS (*n* = 17) and extremity/truncal undifferentiated pleomorphic sarcoma (*n* = 10).	DDLPS: neoadjuvant nivolumab 3 mg/kg or nivolumab 3 mg/kg + ipilimumab 1 mg/kg. Undifferentiated pleomorphic sarcoma: nivolumab 3 mg/kg + radiation or nivolumab 3 mg/kg + ipilimumab 1 mg/kg + RT.	Pathologic response (percent hyalinization): DDLPS = 8.8%. Undifferentiated pleomorphic sarcoma = 89%.	Suggestive of sensitivity of resectable undifferentiated pleomorphic sarcoma to combination of immunotherapy plus RT
SARC032 (Mowery *et al*. 2024)^91^	Grade 2–3 extremity/limb girdle undifferentiated pleomorphic sarcoma or DDLPS or pleomorphic liposarcoma (*n* = 127).	Preoperative RT then surgery *versus* preoperative pembrolizumab (3 cycles) + RT + surgery + adjuvant pembrolizumab (14 cycles).	Improved DFS with administration of pembrolizumab (HR 0.61, 90% c.i. 0.39, 0.96). Absolute improvement of 15% in 2-year DFS with pembrolizumab (67% *versus* 52%). Greater effect seen in grade 3 *versus* grade 2 disease.	Addition of perioperative pembrolizumab to preoperative RT and surgery in resectable undifferentiated pleomorphic sarcoma improves DFS. Long-term results (OS) pending.

eSTS, extremity soft tissue sarcoma; RT, radiation therapy/radiotherapy; DFS, disease-free survival; OS, overall survival; IGRT, image-guided radiation therapy; SABR, stereotactic ablative radiotherapy; RCT, randomized clinical trial; OR, odds ratio; c.i., confidence interval, HR, hazard ratio; i.v., intravenously; DDLPS, dedifferentiated liposarcoma.

Alternative RT delivery methods, such as brachytherapy, have also been evaluated. Pisters *et al.*^74^ randomized 164 patients with STS to adjuvant brachytherapy (iridium-192, 42–46 Gy over 4–6 days) or no further treatment after complete resection, demonstrating improvements in local control, limited to patients with high-grade disease (5-year local control 89% with adjuvant brachytherapy *versus* 66% without). Brachytherapy, when utilized as monotherapy, is currently limited to low-risk cases (for example, small-to-mid-sized high-grade tumours) or for re-irradiation (in order to minimize normal tissue toxicity); it is not routinely considered for high-risk deep-seated tumours^92^.

Although the above prospective trials firmly established the role of adjuvant RT in optimizing local control after limb-sparing surgery and set 5-year local recurrence rates < 10% as the benchmark for future studies, several potential benefits of neoadjuvant treatment have increasingly been recognized. Adjuvant RT is usually administered as 50–50.4 Gy in 1.8–2 Gy fractions, with an additional 10–16 Gy boost (total 60–66 Gy) targeted to the tumour bed and volume at risk of local recurrence^4,6,93^. In contrast, neoadjuvant RT allows for lower doses, typically 50 Gy with no boost, administered to a smaller volume, as the *in situ* tumour facilitates accurate delineation of a tumour and the at-risk region for RT planning. Preoperative RT can effectively render a tumour non-viable and, in select cases, downsize tumours^94^, particularly important in borderline resectable cases. Finally, for planned R1 resections, preoperative RT may sterilize tumour margins, offsetting the impact of a positive margin^95^.

Preoperative *versus* postoperative RT was evaluated in the landmark National Cancer Institutes of Canada NCIC-SR2 trial^72^, in which patients with extremity STS were randomized to 50 Gy RT in 25 daily fractions given before surgery or to 66 Gy in 33 fractions after surgery. In the preoperative group, those with positive margins also received a postoperative boost of 16–20 Gy. No differences in OS or local control were seen, a finding subsequently replicated by others^96^. However, the toxicity profiles of preoperative and postoperative RT differed widely. Preoperative RT was associated with higher rates of major wound complications (35.2% preoperative RT *versus* 17.0% postoperative RT), which included second operations or invasive procedures, deep packing, or admission to hospital for wound care^72^. These occurred almost exclusively in patients with STS of the lower extremity (1 major wound complication reported in the upper extremity), more often in the thigh than the lower leg. However, longer-term results demonstrated a higher rate of late toxicity among those who received postoperative RT^73^, including high rates of grade ≥ 2 fibrosis (48.2%), joint stiffness (23.2%), and oedema (23.2%), with toxicity rate associated with higher dose, larger RT volume, and poorer functional outcome. In addition, the application of an additional postoperative boost to those who received preoperative RT did not improve local control.

Further advances in surgery and RT have aided in reducing morbidity and improving functional outcomes. Intensity-modulated radiotherapy (IMRT), which utilizes multiple beams of varying intensity to provide greater dose conformality and tumour coverage, was shown to provide comparable or slightly improved local control compared with 3D-conformal radiation therapy (3DCRT), with lower rates of late toxicity, including a 12% rate of fibrosis at 2 years^97–99^. Further to this, image-guided radiation therapy (IGRT) utilizes pretreatment MRI allowing for at-risk margin adaptation of the target volume and assessment of treatment CT imaging to ensure accurate, reproducible, patient setup and treatment delivery. IGRT was tested in the phase 2 Radiation Therapy Oncology Group RTOG-0630 trial^75^, in which 10.5% of patients treated before surgery with image-guided (IG)-IMRT or IG-3DCRT developed at least one grade ≥ 2 late toxicity, compared with 37% in the NCIC-SR2 trial. Wound complication rates after preoperative RT to the lower extremity remained high, as also seen by O’Sullivan *et al*. (2013)^100^, where 30.5% of patients who received preoperative IG-IMRT developed significant wound complications.

Given the comparable efficacy but higher rates of long-term, often permanent, toxicities of postoperative RT, many societies have endorsed the use of preoperative image-guided IMRT-based RT for extremity STS^4,5,93^. However, this decision remains an individualized one, taking account of specific patient, tumour, and technical factors. The common exception for preoperative RT are patients who present with a rapidly growing symptomatic tumour who would be unable to tolerate preoperative RT. Tumour location is integral to decision-making, particularly when critical surrounding structures may be at risk from postoperative RT (for example, brachial plexus), or lower extremity location results in high risk of acute complications from preoperative RT. Borderline tumours, where downsizing will facilitate resectability, stand to benefit from preoperative treatment, which has been shown to reduce tumour volumes by a median of 33%^94^. Downsizing can be safely attempted with RT alone, particularly in myxoid/round cell liposarcoma, or considered with sequential or concurrent chemotherapy^101^. Finally, preoperative RT may also contribute to reducing viability of tumour cells and so should be used in all cases where an R1 resection is planned for preservation of critical structures^95,102^.

Beyond the decision of timing of RT, an additional consideration is whether low-risk groups can be identified in whom RT can be safely omitted. In a retrospective study^103^ of patients with mostly small and low-grade extremity and truncal STS, 10-year local control without RT was > 90%. These results were further confirmed prospectively in a trial of selective use of postoperative RT, where RT was omitted in patients with extremity or truncal STS ≤ 5 cm unless pathology demonstrated a positive margin^104^. At 5 and 10 years, local recurrence in the group where RT was omitted remained favourable (7.9% and 10.6%, respectively), suggesting safety of omission in this low-risk group. Based on this, a more selective approach is now undertaken, considering tumour size, grade, histology, and margins, such that RT is omitted for small (< 5 cm), low-grade, superficial tumours^5,6^. Published nomograms can further assist in identifying patients at low risk for recurrence who may be spared from RT^105,106^. In the setting of higher-grade or larger tumours, retrospective data suggest that an approximately 90% local control rate is attainable without RT when patients are treated by expert sarcoma surgeons at high-volume centres^103,107^.

An emerging area of investigation in extremity STS is the use of hypofractionated preoperative RT regimens. Acknowledging that the conventional 5-week course of radiation can be costly and difficult for patients, especially those living far from a specialist treatment centre, an inefficient use of hospital resources, and prolongs the time from diagnosis to surgery, moderately hypofractionated and ultra-hypofractionated regimens, which deliver larger daily doses of RT over a shorter duration, are attractive. Preoperative moderately hypofractionated RT (15 fractions of 2.85 Gy over 3 weeks, total dose 42.75 Gy) has shown promising 30-month local recurrence-free survival (LRFS) of 93% and major wound complication rates of 30.8% in a single phase II trial^76^. Recent longer-term follow-up from this study demonstrated a 4-year LRFS of 93%. Nine percent of patients had grade ≥2 toxicity at 2 years, including skin toxicity (2%), fibrosis (2%), oedema (3%), and joint stiffness (1%). Four patients (3%) experienced bone fractures, all of which occurred in the femur^77^. The ongoing SCOPES phase II trial (NCT04425967)^108^ seeks to explore further the oncologic outcomes, toxicity, health-related quality of life, and cost-effectiveness associated with a moderately hypofractionated regimen (14 fractions × 3 Gy) in patients with intermediate- and high-grade localized STS.

Preoperative ultra-hypofractionated RT has also been evaluated in several studies, delivering 5–8 Gy per fraction daily over 1–2 weeks. When the biologically equivalent dose is below that used in conventional fractionation (for example, 5 Gy × 5 fractions, biologically equivalent dose 37.5 Gy), local control is compromised^78^. However, when doses are similar to or higher than conventional RT (for example, 6 Gy × 5 fractions, biologically equivalent dose 50Gy^79,82^; 7 Gy × 5 fractions, biologically equivalent dose 64Gy^81^; or 8 Gy × 5 fractions, biologically equivalent dose 80Gy^80^), local control reaches > 90%. Acute wound complications rates remain similar to those observed in the NCIC-SR2 trial. Lower rates of long-term toxicity, including fibrosis, oedema, and joint stiffness, are observed but emerging long-term data suggest the possibility of higher rates of amputation for treatment-related complications (up to 16%^80^) and late severe skin toxicity (up to 14%^79^). As data mature, follow-up on long-term toxicities of ultra-hypofractionated regimens will require further evaluation, particularly for those regimens with higher biologically equivalent doses than conventionally fractionated RT. The current consensus is to continue with conventional fractionation and consider hypofractionated schedules in the context of an RCT^109^.

#### Chemotherapy

The use of chemotherapy in the treatment of extremity STS is not routinely recommended due to a conflicting evidentiary base marked by differences in regimens used and results obtained, *Table 2*. However, several guidelines^5,6^ encourage individualized treatment, including consideration of chemotherapy for patients with high-risk disease. The first generation of trials evaluating the efficacy of chemotherapy for STS were meta-analysed in the 1997 review by the Sarcoma Meta-analysis Collaboration (SMAC)^110^. This individual patient data meta-analysis included 1568 patients with mixed histologies and anatomic locations of STSs across 14 randomized trials. Adjuvant doxorubicin-based chemotherapy was shown to improve rates of local recurrence (hazard ratio (HR) 0.73, 95% confidence interval (c.i.) 0.56 to 0.94; absolute risk reduction (ARR) 6% at 10 years) and distant recurrence (HR 0.70, 95% c.i. 0.57 to 0.85; ARR 10% at 10 years) but did not result in a statistically significant improvement in OS (HR 0.89, 95% c.i. 0.76 to 1.03; ARR 4% at 10 years). Subgroup analyses suggested largest benefits among patients with extremity STS, where a 7% absolute benefit in OS was observed.

In the 1990s, a second generation of randomized trials, which included combination treatment of dose-intensified doxorubicin with ifosfamide, emerged. Of note, only one study in the original SMAC meta-analysis administered this combination. An updated meta-analysis^83^ was therefore performed, adding four studies in which the combination of doxorubicin plus ifosfamide was tested. This meta-analysis again demonstrated improvements in rates of both local and distant recurrence, but also demonstrated an improvement in OS, greatest among studies utilizing doxorubicin plus ifosfamide (HR 0.56, 95% c.i. 0.36 to 0.85; ARR 11%). Although encouraging, the benefits observed were tempered by the added toxicity of combination therapy.

Further concerns about the utility of chemotherapy for STS arose following the publication of the results of the EORTC 62 931 trial, a randomized study^84^ of five cycles of adjuvant dose-intense doxorubicin and ifosfamide administered to patients with grade 2–3 resected STSs of any site. No differences in relapse-free survival or OS were observed, questioning its use among this patient group. However, subgroup analyses performed in this trial, as well as in previous meta-analyses, suggested larger effects of chemotherapy among patients with high-risk extremity STS. In a *post hoc* analysis of the EORTC 62 931 trial, Pasquali *et al*. (2019)^85^ stratified patients by risk, assessed using the Sarculator nomogram^111^, and demonstrated improvements in DFS (HR 0.49, 95% c.i. 0.28 to 0.85) and OS (HR 0.50, 95% c.i. 0.30 to 0.90) among high-risk patients (defined as Sarculator-determined 10-year predicted probability of survival < 60%) treated with chemotherapy. A retrospective study^112^ of 5683 patients similarly demonstrated improvements in OS with the administration of combination anthracycline and ifosfamide-based chemotherapy that was limited to patients with high-risk disease (PERSARC-predicted 5-year OS < 65.8%). In neither study were significant benefits observed when chemotherapy was administered to lower-risk patients, supporting selective rather than routine use of chemotherapy for extremity STS.

With support for chemotherapy building, the benefits of neoadjuvant chemotherapy, including the ability to evaluate *in situ* tumour response and provide early treatment of undetected disseminated disease, were increasingly being acknowledged and studies^113,114^ moving chemotherapy into the preoperative setting were conducted. Further to this, efforts to optimize dose regimens and intensity were undertaken, including the ISG-STS 1001 trial^86,87^. In this study, patients with high-grade, ≥ 5 cm, deep STS of the extremity or trunk were randomized to three cycles of epirubicin plus ifosfamide or a regimen tailored to histologic subtype (high-grade myxoid liposarcoma, leiomyosarcoma, malignant peripheral nerve sheath tumour, or UPS). Unexpectedly, patients treated with a histotype-tailored neoadjuvant chemotherapy regimen demonstrated significantly worse DFS and OS compared with those treated with standard epirubicin and ifosfamide. Although discouraging, this trial provides important prospective data on the efficacy of standard chemotherapy. As was done previously, patients enrolled in this trial were also subsequently stratified by Sarculator-predicted 10-year OS, again demonstrating an improvement in OS restricted to high-risk patients (that is, 10-year predicted OS < 60%) treated with standard chemotherapy (5-year Sarculator-predicted OS 58% *versus* study-observed OS 66%; *P* = 0.04)^115^.

Based on these studies, current recommendations for chemotherapy in extremity STS include individualized use, considering tumour size, grade, histologic subtype, and patient performance status and comorbidities. Among patients considered to be at high risk for recurrence and death, such as those with Sarculator-predicted 10-year OS < 60%, neoadjuvant chemotherapy may be administered either alone or concurrently with RT^101^. Response among patients treated with neoadjuvant chemotherapy should be tracked to ensure absence of progression^5,6^.

#### Immunotherapy

A developing area of investigation in the treatment of STS is the utility of immune checkpoint blockade, also reported in *Table 2*. Despite sarcoma being a relatively non-immunogenic tumour, 18% of patients with metastatic/unresectable STS treated with pembrolizumab in the SARC028 trial demonstrated objective responses^88^. Greatest efficacy was observed among patients with UPS and DDLPS (objective response rate (ORR) of 23 and 10%, respectively, in the expansion cohort^116^). Subsequent correlative analyses demonstrated an association between baseline PD-L1 expression, as well as density of tumour-associated T-cell infiltrates, with immunotherapy response^117^. Further studies^118,119^, including gene expression profiling of STS and the phase 2 PEMBROSARC trial, have demonstrated an association between the presence of intratumoural tertiary lymphoid structures and response to pembrolizumab, suggesting its use as a biomarker for immunotherapy response. Of note, however, patient cohorts in early studies of immunotherapy may have included patients with undifferentiated or dedifferentiated melanoma, which can lack expression of melanocytic markers by immunohistochemistry and be misclassified as UPS^120^—such patients demonstrate robust responses to immunotherapy, akin to other melanoma patients, possibly contaminating the results of initial studies.

Alternate immune checkpoint blockade regimens have shown variable success. For example, in the Alliance A091401 trial^89^, patients with metastatic/unresectable STS treated with nivolumab monotherapy exhibited only a 5% ORR. Although the ORR increased to 16% when combination nivolumab plus ipilimumab was administered, serious treatment-related adverse events also occurred at higher rates compared with monotherapy (26% *versus* 19%, respectively).

The finding in the advanced STS population of immunosensitivity of UPS and DDLPS, as well as the observation of synergistic effects of RT with immunotherapy^121^, has ignited interest in evaluating the role of combination RT plus immunotherapy as neoadjuvant treatment for patients with resectable UPS and DDLPS. Analysis of immune infiltrate in UPS of the extremity and trunk in response to RT suggests that RT may change the tumour immune microenvironment and potentially enhance the efficacy of immunotherapy in UPS^122^. In a non-comparative phase 2 study in which patients with extremity/truncal UPS received neoadjuvant 50 Gy RT concurrently with up to four doses of either nivolumab monotherapy or nivolumab plus ipilimumab, Roland *et al.*^90^ demonstrated a median pathologic response (as measured by percent hyalinization) of 89% among patients with UPS. Of note, although all patients completed preoperative RT, completion of all doses of immunotherapy was higher in the monotherapy *versus* combination therapy arm (83% *versus* 50%, respectively), as was the median pathologic response (90% *versus* 62%, respectively). Although the relationship between percent hyalinization and recurrence/survival is unclear, this study demonstrated feasibility of combination treatment.

Compelling data for the use of immunotherapy in select histologic subtypes are presented in the randomized SARC032 trial^91^. In this study, patients with stage III extremity/limb girdle UPS or DDLPS were randomized to neoadjuvant RT followed by surgery with or without the addition of perioperative immunotherapy, comprised of three cycles of neoadjuvant pembrolizumab (before, during, and after RT) and up to 14 cycles of adjuvant pembrolizumab. With a median follow-up of 43 months, patients treated with perioperative pembrolizumab demonstrated superior DFS (HR 0.61, 95% c.i. 0.39 to 0.96; 2-year absolute difference 15%) with no increase in major surgical complications. In subgroup analyses, this effect appeared driven by efficacy in patients with grade 3 disease; the majority of patients (75%) harboured UPS, with few patients with DDLPS (6%) enrolled in this trial, limiting conclusions that can be drawn from subgroup analyses stratified by histologic subtype. Although robust assessment of differences in OS hinge on longer term follow-up, and the efficacy of cytotoxic chemotherapy in lieu of or in addition to immunotherapy remains uncertain, SARC032 establishes the regimen of perioperative immunotherapy in combination with preoperative RT as an option for patients with extremity UPS.

### Treatment of retroperitoneal STS

#### Surgery

Surgery is the mainstay of treatment for retroperitoneal STS, with the initial operation providing the greatest opportunity for cure^5–7^. As with surgery in the extremity, the goal remains complete resection *en bloc* with adherent structures; however, anatomic constraints of the retroperitoneum and pelvis present unique challenges when attempting to achieve this. A thorough understanding of retroperitoneal anatomy and resectional techniques, in-depth imaging review, multidisciplinary discussion with an expert sarcoma team, and involvement of additional surgical specialists, as needed, are critical to the success of surgery for RPS. Improvements in survival observed in recent years^123^ are believed to be partly attributable to refinement in patient selection, highlighting the importance of considering patient, tumour, and technical factors when approaching RPS surgery^124^.

Candidacy for surgery necessarily includes assessment of performance status and comorbidities to ensure patients can withstand the physiologic insult of surgery and potential postoperative complications^7^. When patients are deemed upfront unresectable, this determination is made due to prohibitive performance status in nearly 50% of cases^125,126^.

Technical inoperability is most commonly due to superior mesenteric artery/vein involvement^125^; however, even among expert sarcoma surgeons, opinions vary on criteria for unresectability. For tumours deemed unresectable, consensus opinion should be sought from an experienced sarcoma multidisciplinary team^7^.

Surgical planning additionally requires consideration of histotype-specific tumour behaviour, including risk of local *versus* distant recurrence and primary tumour growth patterns, to which operative approach is generally adapted^7^. In the case of both WDLPS and DDLPS, local progression is the primary mechanism of disease-related death^127^ and, as such, techniques aimed at reducing local recurrence are paramount. Whereas one surgical approach includes resection of the lipomatous mass *en bloc* with only those structures suspected of being directly invaded, margins between WDLPS and normal retroperitoneal fat can be ill-defined. Additionally, histopathologic organ invasion can be present in more than a quarter of cases where it is not suspected during surgery^128^ and infiltration of capsule/serosa/fascia can be present in almost 40% of cases^129^.

In 2009, two retrospective studies^130,131^ presented data arguing in support of more extensive surgery in the frontline setting. Borrowing from the approach in extremity STS, in which a wide resection with a margin of uninvolved muscle is recommended, the idea of compartmental resection was introduced for RPS. As the margins of retroperitoneal tumours often abut viscera rather than muscle, this approach involves *en bloc* resection of uninvolved surrounding organs and soft tissue—most commonly ipsilateral colon, kidney, partial psoas muscle, and peritonectomy—to maximize the ability to achieve R0 resections along these surfaces. Marginal excision is still performed along critical structures that are not frankly invaded, such as the duodenum, pancreas, major vessels and bony structures, as is done in the extremity. The original reports of this technique demonstrated significant reductions in local recurrence with extended surgery^130,131^, in one series reaching as low as 10% local recurrence at 3 years^131^. A follow-up study^132^ additionally demonstrated improvements in survival for patients with grade 1–2 retroperitoneal STS, a group among whom rates of distant recurrence are low and so benefits of reducing local recurrence would be expected to be most pronounced.

**Fig. 2:**
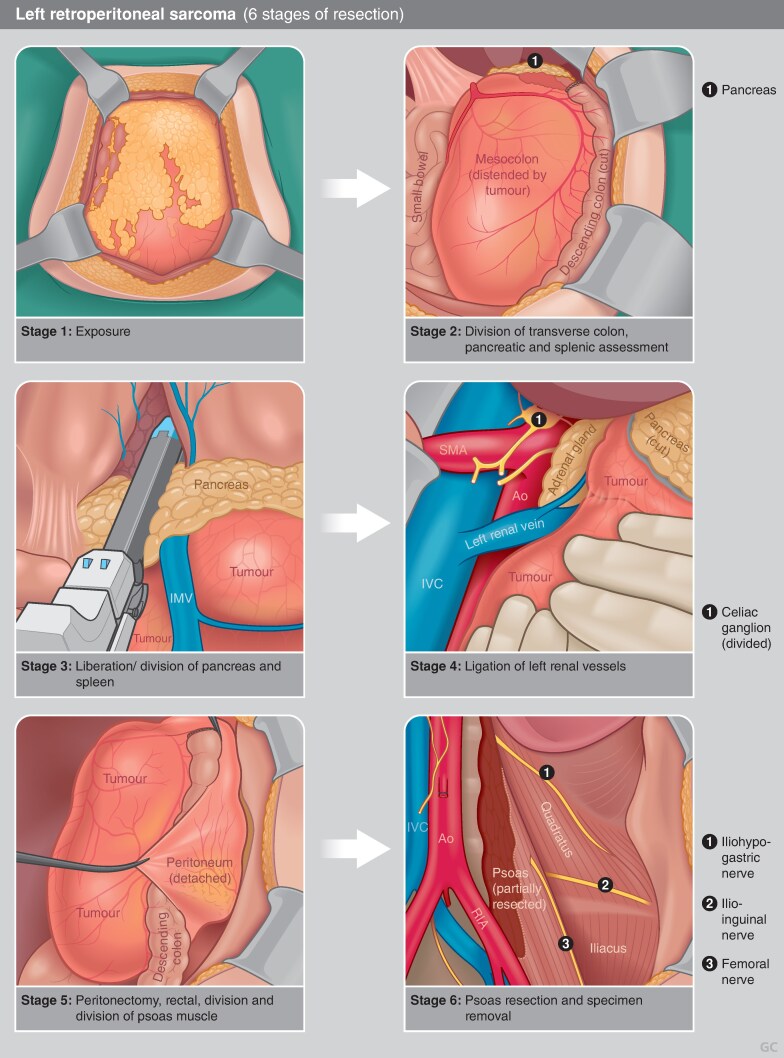
Six stages of resection of left-sided retroperitoneal sarcoma **a** Stage 1: exposure—involves laparotomy with standard xipho–pubic incision and lateral, subcostal, or inguinal split, as required. **b** Stage 2: division of transverse colon, pancreatic and splenic assessment—the transverse colon is divided, ideally distal to the middle colic vessels. The body/tail of the pancreas and spleen are also assessed at this time. **c** Stage 3: liberation/division of the pancreas and spleen—if not involved, the pancreatic body/tail and spleen are medialized; distal pancreatectomy and splenectomy are required in 40–50% of cases due to tumour adherence/involvement. **d** Stage 4: ligation of the left renal vessels—the aorta is cleared of fatty tissue and the left renal vessels are isolated and divided. **e** Stage 5: peritonectomy, rectal division, and division of the psoas muscle—peritonectomy is performed. The rectum is next divided. After identification of the femoral nerve, the psoas is additionally divided in the pelvis. **f** Stage 6: psoas resection and specimen removal—the psoas muscle is resected along the spine, sparing the femoral nerve roots and iliohypogastric nerve. The costodiaphragmatic fold is liberated and the specimen is freed and removed.

**Fig. 3:**
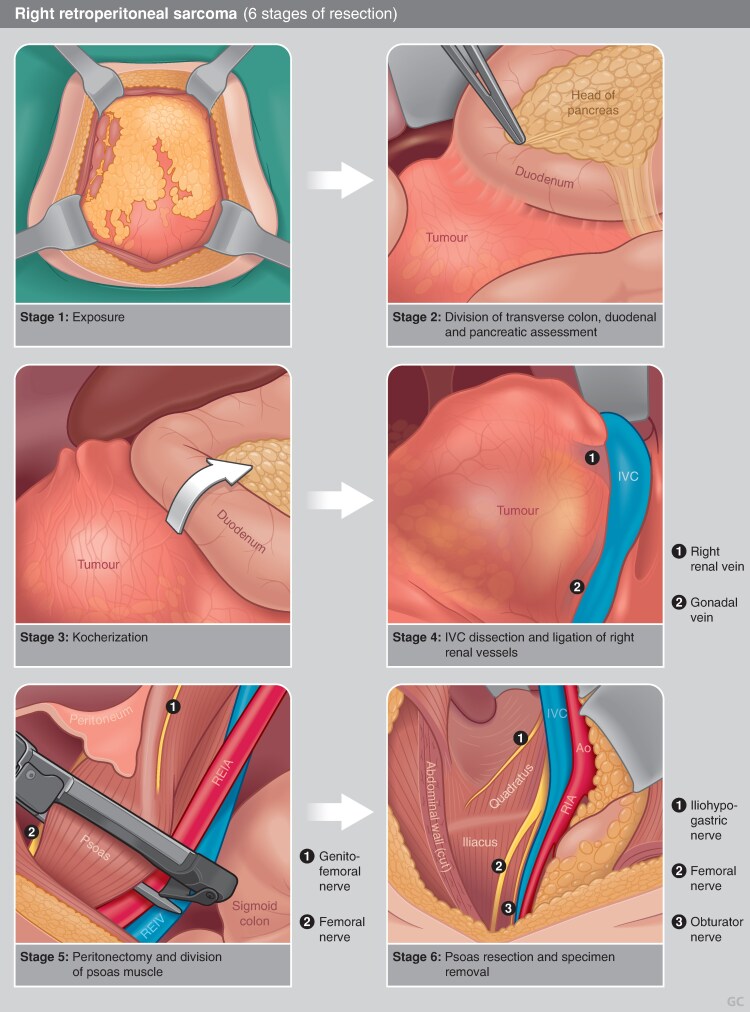
Six stages of resection of right-sided retroperitoneal sarcoma **a** Stage 1: exposure—involves laparotomy with standard xipho–pubic incision and lateral, subcostal, or inguinal split, as required. **b** Stage 2: division of the transverse colon; duodenal and pancreatic assessment—the transverse colon is divided, ideally proximal to the middle colic vessels. The distal ileum is resected with division of the ileocolic vessels. The duodenum and pancreas are assessed for involvement. **c** Stage 3: Kocherization–Kocher manoeuvre is performed. Duodenal dissection is performed and the posterior pancreas is liberated off the tumour. **d** Stage 4: inferior vena cava dissection and ligation of right renal vessels—the inferior vena cava is cleared of fatty tissue. The right renal vessels are isolated and divided. **e** Stage 5: peritonectomy and division of the psoas muscle—peritonectomy is performed. The iliac vessels are dissected and the psoas is additionally divided in the pelvis. **f** Stage 6: psoas resection and specimen removal—the psoas muscle is resected along the spine, sparing the femoral nerve roots and the iliohypogastric nerve. The costodiaphragmatic fold is liberated and the specimen is freed and removed.

The approach of resecting uninvolved organs to improve local control has been criticized for several reasons, including concerns about the biases inherent to retrospective studies, the postoperative morbidity and functional consequences of these extensive resections, an inability to replicate the low rates of local recurrence seen in initial studies, and the observation that recurrences can be multifocal and sometimes outside the initial operative field^133–137^. Whereas prospective data to settle this debate are unlikely to be collected, current guidelines support macroscopic tumour resection *en bloc* with involved organs, including consideration for extended resection to adjacent but not overtly invaded organs for retroperitoneal liposarcoma^5–7^. Codification of the steps of compartmental resection for liposarcoma aims to standardize this procedure among sarcoma experts^138,139^ (*Figs 2* and *3*); however, decisions regarding organ resection or preservation should continue to be individualized^6,7^.

In tandem with technical factors, surgical planning requires consideration of the cost inherent to resection in terms of potential postoperative morbidity. Severe postoperative morbidity from RPS surgery is reported in the range of 16 to 30%, with 10–15% of patients requiring reoperation^136,140,141^. Postoperative morbidity is influenced by the number and types of organs removed, with morbidity increasing when greater than three organs are resected^142^, or resection entails pancreaticoduodenectomy, vascular resection, or combined resection of colon, kidney, spleen, and pancreas^140^. The recently developed Surgical Complexity Score, which incorporates patient factors and resection patterns (that is, resection of lower- *versus* higher-risk organs), can be used to predict postoperative morbidity for patients undergoing resection of primary RPS^143^. Additionally, malnutrition, which can be present in over 50% of patients with RPS, is associated with reduced survival and higher rates of postoperative complications^144–146^. A structured perioperative nutritional rehabilitation programme can improve these rates^147^. As such, nutritional assessment should be performed early and enteral or parenteral supplementation provided for at least 2 weeks before surgery and in the early postoperative period, as needed^7^. Benchmark values for postoperative outcomes among low-risk patients undergoing resection of primary retroperitoneal liposarcoma are now available^148^. The benchmark value for major complications among such patients is ≤ 21% and can be used by institutions for audit and quality improvement.

Longer-term complications have also been evaluated, though to a lesser degree. Nephrectomy, such as performed routinely for compartmental resection, is associated with reductions in the estimated glomerular filtration rate; however, development of stage 4 or 5 chronic kidney disease and need for dialysis are rare^141,149^. Long-term functional consequences are understudied; however, existing data demonstrate 12-month postoperative global quality of life to be comparable to the general population, with minor decrements in physical/lower limb function. Chronic pain, when present, is usually of low intensity. Neuropathic pain, associated with psoas muscle resection, can occur with frequency but necessitates pharmacologic treatment in only 5% of patients at 1 year^141,150^.

Whereas compartmental resection is considered in cases of liposarcoma, a more selective approach to organ resection is recommended for histologies where the primary pattern of failure is distant disease^5–7^ and borders more clearly defined, such as leiomyosarcoma. In such cases, surgery should involve complete macroscopic resection *en bloc* with involved organs (including vein of origin), with preservation of adjacent but uninvolved structures^7,151^. These principles hold true for other histologies with similarly well defined borders and low rates of local recurrence, such as solitary fibrous tumours.

#### Radiation

Several studies have evaluated the role of RT in the treatment of RPS. Whereas intraoperative RT, brachytherapy, and adjuvant external-beam RT are of unclear benefit and are associated with significant short- and long-term toxicities^152–165^ neoadjuvant RT remains among the adjunctive therapies for RPS. Compared with adjuvant RT, when administered before surgery, a lower dose is delivered and radiosensitive organs (for example, small bowel) are displaced by tumour, reducing their RT exposure and related toxicity, thereby improving the risk–benefit balance.

Prospective evaluation of the efficacy of preoperative RT for retroperitoneal STS has proven challenging. ACOSOG Z9031 (NCT00091351), a National Cancer Institute phase III trial, which randomized patients with retroperitoneal or pelvic STS to surgery with or without preoperative RT, was closed early due to slow accrual. The EORTC STRASS trial^166^ is the first study to provide robust prospective data informing the role of preoperative RT for retroperitoneal STS. This phase III trial randomized patients with operable retroperitoneal STS to preoperative RT and surgery *versus* surgery alone; the primary outcome was abdominal recurrence-free survival (ARFS). With a median follow-up of 43.1 months, there was no improvement in ARFS with administration of preoperative RT (HR 1.01, 95% c.i. 0.71 to 1.44). Although not powered for subgroup analyses, in a *post hoc* exploratory analysis limited to patients with liposarcoma (where preoperative progression on RT was not included as an event for patients still able to undergo macroscopically complete resection), RT was associated with improvements in ARFS (HR 0.64, 95% c.i. 0.40 to 1.01; 3-year ARFS 71.6% with RT *versus* 60.4% without RT). To evaluate better the utility of RT in the liposarcoma subgroup with adequate power, in a separate *post hoc* analysis, data from patients enrolled in STRASS were combined with data from patients who were eligible but not enrolled (STREXIT cohort)^167^. This combined STRASS and STREXIT cohort analysis again demonstrated a benefit to preoperative RT for patients with liposarcoma. The improvement in local control was limited to patients with WDLPS and grade 1 and grade 2 DDLPS (HR 0.63, 95% c.i. 0.40 to 0.97); preoperative RT was not associated with improvements in ARFS for patients with high-grade DDLPS or leiomyosarcoma. Based on these results, preoperative RT can be considered for patients with WDLPS and grade 1 and grade 2 DDLPS, balancing reductions in late relapse with long-term RT-related toxities^5,7^. However, given the propensity for late recurrences in patients with WDLPS , long-term data are awaited to determine the durability of the effects observed in STRASS and to assess whether survival differences emerge.

#### Chemotherapy

Evidence pertaining to the use of chemotherapy in RPS is generally sparse, with no proven benefits demonstrated for adjuvant chemotherapy in this setting. Two ongoing trials evaluating the role of neoadjuvant chemotherapy are noteworthy. Given the significant risk of distant disease among patients with high-grade DDLPS and leiomyosarcoma, the ongoing EORTC STRASS2 randomized trial (NCT04031677)^168^ is designed to evaluate the efficacy of neoadjuvant histology-tailored chemotherapy for patients with resectable grade 3 DDLPS and leiomyosarcoma. The multi-arm phase I-II TRASTS trial (NCT02275286) will additionally assess the safety and activity of neoadjuvant trabectedin with concurrent RT in patients with upfront resectable retroperitoneal liposarcoma and leiomyosarcoma. Both trials are expected to be completed in 2028. In the interim, outside of the clinical trial setting, neoadjuvant chemotherapy may be considered for the purpose of cytoreduction in patients with borderline resectable disease^7^; however, such treatment decisions should be individualized.

### Locoregional adjuncts for the treatment of STS

Whereas surgery remains the basis for the curative treatment of localized STS, several locoregional therapies have been investigated as adjuncts or alternatives to surgery for the purposes of improving surgical or oncologic outcomes or as bridging or definitive treatments.

#### Transarterial embolization

Minimally invasive endovascular and percutaneous techniques offered by interventional radiology can further contribute to the comprehensive treatment of patients with STS. In the setting of resectable but hypervascular tumours, preoperative devascularization can be achieved via transarterial embolization (TAE). By occluding tumour-feeding arteries, this technique is shown to reduce the need for intraoperative blood transfusion and can potentially reduce tumour bulk^169^. Although not often needed, preoperative TAE can be beneficial in select cases with challenging anatomy where early vascular contrast is documented and targetable.

#### Isolated limb perfusion

For patients with unresectable extremity STS, isolated limb perfusion (ILP) is a locoregional method aimed at avoiding or delaying amputation^170^. This technique involves obtaining surgical vascular access to the limb for the purpose of delivering hyperthermic chemotherapy via extracorporeal circulation. Its efficacy in STS is enhanced by combining melphalan with tumour necrosis factor-alpha (TNF-α); however, broad use is currently restricted by the limited availability of TNF-α. ILP must be performed in specialized centres due to the technical complexity of the procedure and need for leakage monitoring^171^. Currently accepted indications for its use include extremity STS not amenable to limb-sparing surgery, multifocal or multiply recurrent tumours, and locally recurrent tumours in previously irradiated fields. A more recently developed alternative of isolated limb infusion offers a less invasive alternative; however, efficacy data remain preliminary.

#### Regional hyperthermia

Regional hyperthermia (RHT) combined with chemotherapy or RT has been prospectively evaluated as a treatment strategy for high-risk STS of the extremities and retroperitoneum. The EORTC 62961-ESHO 95 trial, which randomized patients with localized, high-risk extremity and retroperitoneal STS (defined as ≥ 5 cm, grade 2 or 3, deep to fascia) undergoing local therapy (surgery +/− adjuvant RT) to perioperative chemotherapy with or without the addition of RHT. Patients treated with RHT exhibited superior local progression-free survival, DFS, and OS that persisted in analyses limited to patients with macroscopically completely resected abdominal/retroperitoneal STS^172–174^. Despite positive data, this approach has not gained widespread consensus within the sarcoma community and remains a subject of debate. Its clinical use is further restricted by the scarcity of dedicated RHT facilities, limiting access to this modality in most centres.

#### Electroporation

For palliation of superficial tumours not amenable to curative therapies, percutaneous electroporation can be used to enhance local drug delivery. This technique utilizes electric pulses to permeabilize transiently tumour cells, markedly increasing cytotoxicity of chemotherapy. In the recent InspECT study of patients with advanced cutaneous angiosarcoma, electrochemotherapy yielded an 80% ORR (40% complete responses) with manageable grade 3 skin ulceration or pain; bleeding was controlled in 13 of 14 patients^175^. However, limited data are available for this technique.

### Future challenges

The early closure of several RCTs investigating therapies for STS highlights the challenge in adequately powering and recruiting patients to trials of novel treatments in localized STS. Whereas histotype-specific and biomarker-driven studies are highly desirable and are most likely to demonstrate the benefits of novel therapeutic approaches, the rarity and ultra-rarity of sarcoma subtypes, and the low frequency of biomarkers, hinder the ability to keep pace with discoveries in other cancer types. However, several lessons can be gleaned from previous experiences. Namely, the success of the STRASS trial underscores the importance of international collaboration through organizations such as TARPSWG, as few institutions globally see sufficient sarcoma volume to run local trials adequately. Future studies that seek to improve trial efficiency and the likelihood of sufficient patient accrual will additionally require novelty not only in therapeutic approaches but also in study design and execution. This can potentially be achieved through the adoption of emerging methodologies, such as adaptive platform trial designs for earlier evaluation of treatment efficacy or futility, and incorporation of robust synthetic controls, such as through digital and molecular twin technologies. The success of these endeavours will rely on partnership with regulatory agencies to explore jointly scientifically acceptable approaches to the study of this rare cancer in ways that do not compromise the certainty in the efficacy and safety of new treatments or unnecessarily impede progress.

## Conclusion

Individualized treatment of STS requires careful consideration of anatomic location, tumour grade, histologic subtype, and patterns of local and distant failure. Surgery remains the cornerstone of treatment for both localized extremity and retroperitoneal STS; however, deliberate integration of neoadjuvant and adjuvant therapies, including systemic and locoregional treatments, can facilitate the goal of cure. Optimal outcomes are achieved when patients are treated at high-volume centres by dedicated multidisciplinary teams. Considering the rarity of this diagnosis, international collaborative efforts remain crucial to ongoing progress in diagnosis and treatment.

## Data Availability

Not applicable.
